# Adolescents’ understanding of the Nepalese version of HLS-CHILD-Q15: qualitative pre-testing in ninth-graders

**DOI:** 10.1186/s12889-024-18329-9

**Published:** 2024-03-19

**Authors:** Shanti Prasad Khanal, Chitra Bahadur Budhathoki, Bhimsen Devkota, Torsten Michael Bollweg, Orkan Okan

**Affiliations:** 1 Faculty of Education, Tribhuvan University, Kathmandu, Nepal; 2 Bournemouth University, St Paul’s Lane, Poole, UK; 3 TUM School of Medicine and Health, Technical University of Munich, Professorship of Health Literacy, München, Germany; 4 TUM School of Medicine and Health, Department of Sport and Health Sciences, Technical University of Munich, WHO Collaborating Centre for Health Literacy, Munich, Germany; 5 School of Medicine and Health, Center for Health and Medicine in Society, Technical University, Munich, Germany; 6 School of Medicine and Health, Center for Health Promotion in Childhood and Adolescence, Technical University, Munich, Germany

**Keywords:** Adolescents, Health literacy, HLS-Child-Q-15, Nepal, School, Qualitative pretest

## Abstract

**Introduction:**

Research on health literacy is still at an early stage, lacking a dedicated measurement instrument for assessing children’s and adolescents’ health literacy. Such tools are necessary to generate the evidence required for informed intervention development. This study translated the validated German HLS-Child-Q15 into the Nepalese context, creating the HLS-Child-Q22-NEP.

**Methods:**

The research team initially created the HLS-Child-Q22-NEP using an additional item pool. We conducted thirteen one-on-one cognitive interviews with adolescent students from community schools in three districts of Nepal during the pre-test. We employed verbal probing techniques and deductively analysed the interviews based on Tourneau’s model, uncovering four main themes: (1) comprehension (with the two sub-categories: a) item comprehension and b) word comprehension); (2) retrieval; (3) judgement; and (4) response.

**Results:**

Overall, participants responded positively to the HLS-Child-Q22-NEP. However, this study revealed comprehension challenges such as unfamiliarity, misunderstandings, and translation issues. Additionally, the study identified retrieval challenges and poor judgement, indicating limitations in the assessment. Participants experienced varying levels of difficulty with some items, emphasising the need for revised instructions. Subsequent revisions, guided by pretest insights, led to the development of the HLS-Child-Q24-NEP.

**Conclusion:**

The development of the HLS-Child-Q22-NEP is a significant step in addressing Nepali adolescents’ lack of health literacy measurement. Despite its generally positive reception, this study encountered challenges in comprehending the scale, prompting enhancements, and developing the HLS-Child-Q24-NEP. Further research, both qualitative and quantitative, is necessary to evaluate the validity and reliability of the modified items.

**Supplementary Information:**

The online version contains supplementary material available at 10.1186/s12889-024-18329-9.

## Background

Health literacy is crucial for public health and health promotion [1, 2]. The concept encompasses “people’s knowledge, motivation, and competencies to access, understand, appraise, and apply health information” [3]. This understanding also extends to children and adolescents [4]. From a developmental and learning perspective, adolescence is a stage of physical and mental changes [5]. Adolescents undergo social, emotional, cognitive, and bodily changes as they grow up. They also engage in risky behaviour, and establish their health behaviours, attitudes, and beliefs. This makes adolescence foundational for lifelong health [6]. Health literacy can help adolescents manage health information and empower them to critically assess health claims, make healthy decisions, and act on their health and well-being [7]. Fleary and colleagues conducted a systematic review to examine the relationship between adolescent health literacy (AHL) and health behaviour, revealing that higher AHL significantly influences the adoption of healthy behaviors [8]. Thus, adolescence presents significant opportunities to promote health literacy and facilitate the development of health-literate adults [9, 10]. When conceptualising health literacy, it is essential to consider adolescents as a distinct target group, taking into account their everyday realities, preferences, and interests in defining health literacy and related activities [9]. However, there appears to be a lack of a clear definition for AHL [9, 11], making it difficult to operationalise and measure, despite several attempts to develop valid, reliable, and age-adapted tools [10, 12].

In the past decade, four systematic reviews on AHL measurement tools have been published, analysing the field, identifying shortcomings in existing tools, and recommending standards for measuring AHL. Ormshaw et al. [13], Okan et al. [10] and Guo et al. (2018) [12] identified 15, 15, and 29 tools, respectively, using somewhat different search strategies and scopes. A recent review by Khanal and colleagues identified 13 tools for assessing AHL [14]. Recommendations from these reviews emphasise the need to develop AHL measurement tools further [10, 12, 13]:

(i) Tools should be based on a clear definition of health literacy tailored to the age and developmental-appropriate needs of children and/or adolescents.
(ii) All aspects of the definition should be exactly operationalised into the tool.
(iii) The tool should be robustly pre-tested using qualitative interview techniques.
(iv) Quantitative testing for psychometric properties should aim for high reliability and validity, and.
(v) Children or adolescents should be included in developing the tool.

Newer tools to assess AHL are comprehensive and multidimensional in design, including the Health Literacy for School-Aged Children (HLSAC) questionnaire [15] and the Measurement of Health Literacy Among Adolescents Questionnaire (MOHLAA-Q) [16]. These tools have been used in population-based studies in some European countries, e.g., the European Health Behaviour in School-aged Children study [17] and the German Health Literacy among Adolescents study [18]. Another tool, the HLS-Child-Q15, assesses subjective health literacy for younger children [19, 20]. Researchers developed, tested, and validated it for 9- to 11-year-old fourth graders in Germany [19], and it has since led to several translations, e.g., a Dutch version [21], an English version [20], a Portuguese version [22], and a French version [23]. The HLS-Child-Q15 represents the first child adaptation of the European Health Literacy Survey Questionnaire (HLS-EU-Q47) [3], which assesses the generic health literacy of adults 15 years old through a four-point Likert scale. The HLS-EU-Q47, a self-report health literacy questionnaire, measures an individual’s perception of the ease or difficulty of health information tasks related to information seeking, comprehension, evaluation, and use [3]. Utilised in the European Health Literacy Study [24], and its follow-up study HLS-19, conducted by the WHO Action Network on Measuring Population and Organisational Health Literacy (M-POHL) [25, 26], it has gained global recognition with widespread use and adaptation [27].

Using a similar methodology, the HLS-Child-Q15 was developed as the child’s version of the tool in collaboration with the original developers of the HLS-EU-Q47 [28]. To pretest the scale, Bollweg et al. (2020) conducted cognitive interviews with children in focus groups. While most items were understood as intended, comprehension problems arose with some health information tasks (e.g., appraising and applying information) due to a lack of experience, leading to misinterpretation [20]. Participants partly answered based on prior knowledge of the health topic, neglecting the perceived difficulty of information-related dimensions. Some tasks were considered parental responsibilities, causing children to refrain from engagement. Domanska and colleagues (2018) highlighted similar findings in cognitive interviews with adolescents during the development of MOHLAA-Q [29]. These studies revealed misinterpretations and difficulties relating to health information tasks, emphasizing the importance of such cognitive interviews in developing new or adapting existing health literacy instruments. This step is crucial for revealing critical issues and improving the questionnaire.

This study aimed to translate and adapt the HLS-Child-Q15 to the Nepalese context and pre-test the tool qualitatively with Nepali adolescents using cognitive interviews. Although initially developed for primary school children, the HLS-Child-Q15 has been tested and validated in secondary school students up to 18 years [30, 31]. In older cohorts, HLS-Child-Q15 demonstrated good internal consistency for adolescents (overall 0.71 to 0.74) and small but significant convergent validity (*r* =.107; *p* <.001), with low discriminant validity [16]. This suggests it is generally an effective HL tool for use in adolescents. To our knowledge, no prior work has been done on developing a comprehensive self-report instrument to assess the health literacy of secondary school-aged adolescents in Nepal [32–34].

## Methods

### Study design

To develop a Nepalese health literacy measurement tool for adolescents, we adapted and pretested the HLS-Child-Q15. After translating the questionnaire into Nepali, we conducted cognitive interviews (CI) with adolescent students in a school setting to explore how participants interpret the questions and refine the tool accordingly.

### Study participants and sampling

Conducting the study at four community schools across three districts in Nepal: Dailekh, Surkhet, and Banke. We recruited participants from two rural schools in Surkhet and from two urban schools in Surkhet and Banke. The caste system in Nepal includes four main categories: Brahmin, Chhetri, Janajati, and Dalit. To ensure a rural/urban distribution, we tested the tool with adolescent groups exhibiting diverse socio-cultural and socio-economic backgrounds. This was achieved through intentional selection across caste and gender. Our commitment was to achieve an even representation of both rural and urban areas within the chosen districts.

We selected schools within these districts through judegmental sampling, consulting with teachers to choose participants representing different characteristics. Following the recommendation that cognitive interviews involve five to 15 people [35], we recruited *N* = 13 adolescent students for the study. Emphasising the depth and richness of data over a large sample size, the intentional selection of participants across diverse characteristics within the specified districts was considered more valuable for the study’s nature.

The recruitment of adolescents occurred in two stages: initially, we selected nine participants, followed by the addition of four participants in the second stage to expand the data scope. We collected data from 13 participants but refrained from including additional participants due to data saturation.

### Using the HLS-Child-Q15

The original item pool of the HLS-Child-Q15, which underwent quantitative pretesting with German schoolchildren, comprised 26 items [19]. However, during the validity and reliability testing of the scale, only 15 items demonstrated adequate performance in the target group. Notably, the health literacy dimensions (accessing, understanding, appraising, and applying health information) were unevenly covered in the HLS-Child-Q15, with only one item addressing the appraisal of health information. We included all 26 items from the German pretest in the Nepalese study. After a thorough examination within the research group, we included 22 items in the Nepali sample (Table 1). We excluded four items after assessing their suitability for this study. The Nepali language version of the scale is provided in Annex 1.

**Table 1:** Adapted nepali items based on the original item pool of HLS-Child-Q15

Item no.	Based on a scale from “very difficult”, “difficult”, to “easy”, and “very easy”, do not know, how easy or difficult is it for you to…
1	find out how to recover quickly when you have a cold?
2	find out what you can do so that you don’t get too fat or too thin?
3	find out how you can best relax?
4	find out which food is healthy for you?
5	understand when and how you should take your medicine when you are ill?
6	understand what your doctor says to you?
7	understand why you sometimes need to see the doctor even though you are not ill?
8	understand why you need vaccinations?
9	understand what your parents tell you about your health?
10	understand why you need to relax sometimes?
11	judge what helps a lot for you to stay healthy and what does not help much?
12	do what your parents tell you to do so that you can get well again?
13	take your medicine in the way you’re told to?
14	stick to what you have learned in road safety lessons?
15	have a healthy diet?
16	judge what helps or does not help to get rid of a cold?
17	judge the truth of what the doctor tells you in order for you to get well again?
18	judge whether you can trust the media when they warn you about risks to your health?
19	judge whether what may happen to you later if you start smoking is true?
20	judge how where you live (neighborhood, district, street) is connected to your health?
21	judge how your behavior (exercise and diet) is connected to your health?
22	decide when you need to wash your hands?

*Note: 1-15-HLS-Child-Q15, 16 to 22- additional Items from HLS-Child-Q15 development item pool

In the development phase of the HLS-Child-Q22-NEP scale, we built upon the foundation laid by the HLS-Child-Q15 [19, 20]. The HLS-EU-Q47 [3], motivated this endeavour to assess adolescents’ generic health literacy. The theoretical foundation of HLS-Child-Q15 is grounded in the conceptual model of health literacy, defining it as multidimensional. This scale incorporates four dimensions of health literacy: accessing, understanding, appraising, and applying health-related information in three health domains: health care, disease prevention, and health promotion [3]. This structured approach offers a comprehensive framework for evaluating health literacy in HLS-Child-Q22-NEP, with each dimension and domain contributing to a nuanced understanding of individuals’ health literacy.

The Nepalese research team translated and back-translated these items (SPK, CBB, BD), while the German team (OO, TMB) verified whether the back-translated versions still captured the original meaning and wording. The pre-tested version of the Nepalese tool, the HLS-Child-Q22-NEP, comprises four items for finding health information, seven for understanding health information, seven for appraising health information, and four for applying health information.

### Cognitive interviews

CIs should be conducted in the early stage of the questionnaire development process [35] to gain insights from the participants and evaluate their interpretation of the survey questions [36]. In the present study, CIs specifically identified and corrected any underlying semantic, syntactic, cultural, and methodological problems with the items of the HLS-Child-Q22-NEP, ensuring participants understood them as intended. During the CIs, participants received the Nepali version of the HLS-Child-Q22-NEP questionnaires. This procedure aligned with the development process of the HLS-Child-Q15 [19] and another tool adaptation from the HLS-EU-Q47 for adolescents, resulting in the MOHLAA-Q [29].

In this study, we employed verbal probing techniques as a CI method, following the approach suggested by Willis [37] and Meadows [38]. This method involved dividing the questions into two parts prior to the CI to assess the survey items. The first part consisted of all 22 items from the original form, the HLS-Child-Q22-NEP (see Table 1), and we also included additional open verbal probing questions [37]. During the cognitive testing phase, we utilised general, specific, and comprehension questions to investigate and analyse how adolescents comprehend the items and their relevance to their health literacy-related activities [29, 36]. Throughout the interview, we combined both scripted and spontaneous probes [37]. General probing questions included inquiries like ‘*Tell me, how easy are the questions on this scale for you?’ and* ‘*What are the easiest and most difficult questions for you?’* Specific probing questions, as used by Domanska et al. [29], were employed to test participants’ comprehension [37] of specific terms or subject matters (e.g., *‘What were you thinking when you read the term “have a cold?) “’, What can you do to prevent being too thin or too fat?‘)*.

### Data collection procedure

During the data collection, the Nepali principal researcher played a crucial role in conducting all thirteen cognitive interviews with grade nine adolescent students from 19th April to 5th May 2022. Only the first author and selected participants attended the pretest. Following the interview guidelines, we conducted the interviews in the Cognitive Laboratory Environment (CLE) [37]. CLE refers to the classrooms of the respective schools. It provides a welcoming environment for students to participate in CI activities. This is where we conducted CI with students during office hours, with the support of school health and physical education teachers. The interviewers started by introducing the researcher to the whole group and explaining the main purpose of this study. Following that, adolescents were provided with a printed copy of the Nepali survey questions for them to read and reflect upon. We then conducted one-on-one in-person interviews [37] with each individual, which lasted between 43 min and one hour. We audio-recorded the interviews for later analyses. During the interview, we asked additional probing questions to elicit the respondents’ understanding of each point, and they were encouraged to share their views and experiences with the items.

### Data analysis

The rationale for using deductive methods in data analysis is based on established practices and models within the field. This is particularly evident in prior research on survey pre-testing. The decision to analyse the interviews deductively aligns with the widely used model by Tourneau, which has consistently been applied in studies focusing on survey pre-testing [29, 39]. Tourneau’s general model includes comprehension, retrieval, judgment, and response (CRJR), providing a structured and systematic framework for analysing interview data [40]. The deductive approach extends to thematic coding, following the guidance of Meadows [38], which serves to further refine and generate coding categories and subcategories. We rigorously and systematically analysed the data using ATLAS ti software. We transcribed the audio recordings of the interviews into Nepali and then translated them into English. We created separate labels to be applied to sections of the interview data [38]. The results section illustrates respondents’ views on categories and subcategories through the provided excerpts. We used a transliteration method for some specific words expressed by participants. As a final step, we combined the coding to create four main segments: (1) comprehension, (2) retrieval, (3) judgement, and (4) response. The comprehension category combined two subcategories: item comprehension and health term comprehension (superficial and misunderstanding).

Comprehension refers to understanding and interpreting written information, as well as deriving meaning from it by using prior knowledge (e.g., about the grammatical structure, topic, or context of a text) [39]. Retrieval refers to the process of recalling or reconstructing information from memory. Judgement involves using and combining information to form an opinion or estimate, specifically regarding the perceived difficulty of the tasks addressed in the HLS-Child-Q15 questionnaire. Response entails making a choice and selecting one of the provided response categories [40].

### Ethical consideration and data protection

The Nepal Health Research Council (NHRC) reviewed and approved the study protocol (Ref. No. 2688). The research team informed participants and parents about the study aims, participant rights, and data protection procedures. Adolescents participated voluntarily, providing written consent and agreeing to the audio recording of interviews. We assured participants that they could choose not to participate or respond. To maintain confidentiality, the study implemented strict coding measures to ensure the anonymity of participants’ responses. During the COVID-19 pandemic, both researchers and participants followed WHO-recommended protocols [41]. The original authors of HLS-Child-Q15 approved and actively supported the study, reinforcing its ethical integrity.

## Results

### Sample characteristics

The study comprised 13 adolescents enrolled in the ninth grade across four community schools, encompassing urban and two rural areas in three districts (Dailekh, Surkhet, and Banke). The participants consisted of eight females and five males, belonging to various castes and ranging in age from 13 to 19 years (Table 2).

**Table 2:** Personal characteristics of the participants

Students’ ID	School	Age (years)	Sex	Caste	Area
1	1	14	Female	Brahmin	Rural
2	2	13	Female	Brahmin	Rural
3	3	14	Female	Brahmin	Urban
4	3	15	Female	Dalit	Urban
5	4	16	Female	Janajati	Urban
6	2	15	Male	Janajati	Rural
7	1	19	Male	Dalit	Rural
8	4	15	Male	Chhetri	Urban
9	1	16	Male	Chhetri	Rural
10	1	15	Female	Dalit	Rural
11	2	14	Male	Janajati	Rural
12	3	13	Female	Chhetri	Urban
13	4	15	Female	Brahmin	Urban

### Comprehension

#### Items comprehension

Participants generally responded positively, indicating they read and understood the survey questions. While all participants interpreted question 1 consistently, there were notable discrepancies in the interpretation of certain items, such as 2, 8, 14, 16, and 18. Participants encountered problems with the scale’s instructions (“How easy or difficult is it for you to…”) and struggled to link them appropriately to subsequent questions. The analysis suggests that presenting these instructions individually for each item, rather than only once at the top, could significantly enhance comprehension.

Only one participant (ID_09) understood all questions thoroughly when examining the overall questionnaire items. Notably, a female participant (ID_13) found items 1 and 8 very easy, while a male participant (ID_8) reported high confidence in understanding items 19 and 22.

ID_08: *I found questions 1, 6, and 14 difficult, questions 9, 11, 17, 18, and 20 very difficult, and questions 19 and 22 very easy. The rest of the questions seemed easy.*

ID_09: *All the items are easy. There is not one I do not understand.*

Further insights from individual participants’ highlighted varying degrees of perceived difficulty. For instance, most participants thought that item 14 (‘*…stick to what you have learnt in road safety lessons?’*) was challenging to understand. Similarly, five participants deemed items 18 and 22 difficult, indicating potential comprehension issues with these particular questions. On the contrary, only a few participants encountered challenges with items 8, 6, 11, 13, 16, 17, and 20, suggesting a better understanding of these items among the sample. Three participants specifically encountered difficulty with question 11, while very few participants considered items 1, 6, 9, 13, 16, and 17 to be complicated and challenging to understand. Overall, most participants showed a positive attitude towards the survey questions. However, the analysis reveals specific challenges in comprehension, mainly related to certain items and the scale’s instructions.

The analysis showed that participants encountered comprehension challenges related to some items due to inconsistent syntax and vocabulary. For example, using the word ‘cold’ in item 1 proved challenging, as its Nepali translation *(chico/jado)* refers to being cold or icy, rather than the common cold (the disease). Participants grasped the intended meaning only when reminded of the latter. Similarly, difficulties arose with the words *adhik* (too much) and *paatalo* (thin) in item 2. Replacing *paatalo* with *dublo* (with the same meaning) and too much (*adhik with ‘dherai*) led to improved participant understanding. Substituting *khop* for vaccine in item 8 also resulted in better comprehension. Moreover, adding the term “stick to” (*adig*) in item 14 proved challenging due to its unfamiliarity. Some items had mistranslations and inconsistencies, including repetitive words, misplaced adverbs, pronouns, and grammatical flaws in Nepali. Items 1, 5, 6, 11, 12, and 14 exhibited consistent wording, with repeated words and disrupted sentence structures. Pronoun repetition such as “you” *(tapai)*, as observed in items 2, 11, and 21, contributed to confusion, while the inclusion of adverbs like “how” (*kasari*) in item 3 and “why” (*kina)* in items 7, 8, and 10 further hindered comprehension. The lack of conjunction in item 4 made it unclear, and the wording “what do you understand” (ke bhujhnuhunchha) in question 6 introduced vagueness. The repetition of the word “helps a lot” (*dherai maddat)* in item 11 complicated the question. Participants found using the reflective pronoun “for you” (*tapaaiko laagi*) in item 5 to be irrelevant. Using the integrative pronoun “what” *(ke)* in question 9 complicated the question for some. The adjective “you can” *(sakos)* in item 11 posed difficulties for some participants. Additionally, the absence of an adjective in item 14 made the question difficult to understand, and the wording “what happens” *(ke hunchha)* in item 19 was not found to be helpful.

The HLS-Child-Q22-NEP survey faced challenges due to the Nepali grammar rule of *Hraswa Dirgha*, leading to potential alterations in meanings. Violations of *Hraswa Dirgha* were identified in the scale’s instructions, as well as the use of the pronoun “you” (*tapaai*) in item 5. Additionally, inconsistencies in the preposition usage “of” (*ko)* in items 16 and 19, “in” *(ma) in* item 18, were noted, affecting the clarity of participants’ interpretations. Participants highlighted irregular sentence structures in items 7, 13, 18, and 22, suggesting potential confusion and hindrance to comprehension. In summary, the grammatical and syntactic challenges identified suggest a need for a careful revision of certain items to enhance the survey’s effectiveness in capturing health literacy among Nepali teens.

### Health terms comprehension

#### Superficial understanding

The participants generally demonstrated a superficial and only partially correct comprehension of the terms included in the questionnaire items. None of the participants showed familiarity with key health-related terms such as health literacy, health risks, health promotion, determinants of health, and health warning. Adolescents demonstrated a subjective and often superficial understanding of these health terms during interviews, with certain terms proving to be beyond their comprehension. Specifically, the term “cold” in item 1 prompts an interesting observation.

Participants correctly associated it with its literal meaning of “cold or icy” *(chiso or Jaado)*, but when asked about the disease ‘common cold’, they expressed familiarity with it and misconceptions about its causes. This underscores that conveying accurate health concepts to Nepali teens through HLS-Child-Q22-NEP faces challenges, highlighting difficulties in addressing deeper understandings due to participants’ surface-level associations with health terms.

ID_07: *Common cold is a disease caused by eating rotten and cold food, from which a running nose (Hachhyu) comes.*

Despite challenges with health terms, many participants showed familiarity and understanding with the phrase “find out how you can best relax” *(asal aaram)* in item 3. Notably, some linked it to sleeping, recognising it as a way to rest when tired. This suggests a shared and relatively clear understanding of “how to best relax”. The association with sleeping indicates a practical comprehension, implying that certain health-related concepts are more accessible and well-understood in the context of daily life experiences for these adolescents.

ID_10: *When we are tired after working or playing a lot, we can lie on the bed and rest, but when we are a little tired, we can sit on a chair and rest our heads.*

Despite adolescents demonstrating an understanding of the term “healthy food” *(swastha khana)* in item 4, they struggled to provide specific examples when prompted. One participant’s response, suggesting that ‘… *eating favourite food is healthy, reveal*s a limitation in the scale’s ability to assess nuanced understandings of healthy dietary practices. Moving to item 8, which addresses vaccination, all adolescents appeared familiar with the term vaccine *(khop)*. However, the extent of their comprehension is not explored further in terms of specific vaccines or their importance. In response to item 11, which asks about staying healthy, participants primarily mentioned biomedical concepts like hospital visits or taking medication. Surprisingly, participants omitted health promotion or engagement in social health-related activities. These omissions suggest a limitation in capturing a holistic understanding of health by the HLS-Child-Q22-NEP in this sample.

ID_02: *Use of hospital rules and medicine* to stay healthy.

Although participants were familiar with the term *health behaviour* (item 21), there seems to be a gap in understanding its connection to their health and well-being.

ID_09: *A healthy activity such as drinking hot water or cold water when you have a cold, I didn’t know much about it.*

Although most participants demonstrated a basic understanding of the term ‘hand washing’, none could recall the accurate steps and methods, revealing a limitation in their comprehension.

ID_07: *An activity to stay healthy that is done in 9 steps. Hands should be washed before and after eating*.

### Misunderstandings

Participants’ lack of familiarity with the phrase “too thick or too thin” *(dherai moto ra dherai patalo)* in item 2 illustrates misunderstandings. Additionally, some participants perpetuated stereotypes related to body image by believing that they could assess whether someone is thin or thick through visual observation. Only one participant provided a partially correct answer regarding the intended action of item 2, which focuses on managing one’s own body weight. A few of them misunderstood the term vaccine (item 8) as medicine for treatment.

ID_04: *A vaccine is an injectable medicine.*

Furthermore, most participants misunderstood item 14 (“stick to what you have learned in road safety lessons?”) as we intended. They associated “road safety” with activities like cleaning, maintenance, and avoiding road destruction, rather than traffic rules. This highlights the importance of taking into account participants’ local context and living conditions in order to obtain a comprehensive comprehension of health concepts on the scale of this sample.

ID_12: *Road safety measures include pitching the road, putting a line in the middle to reduce the risk of a collision, and driving in your line.*

*ID_04: Maintain the road without dumping garbage, digging, or demolishing.*

A significant portion of participants showed unfamiliarity with the term ‘media’ *(aamsanchar)*, notably highlighted in item 18. The analysis indicates that a considerable number of respondents had a limited understanding of the term. Those who were familiar with it had a limited understanding, perceiving it solely as a social media platform.

ID_04: *Media is the process by which journalists collect news and provide information on events in society.*

Concerning item 18 of the survey, only a few participants demonstrated awareness and understanding of the terms “health warning” *(Swasthya Chetawani)* and “health risk” *(Swaasthya Jokhim)*. In item 19, participants inaccurately grouped distinct terms such as alcohol, drugs, and cigarettes under the same category, despite being familiar with the term ‘smoking’.

### ID_05: *Alcohol, drugs, and cigarettes are known as “smoking.”*

### Retrieval

Although all participants promptly responded to the survey, they exhibited varying levels of recall regarding health-related topics. Some participants encountered challenges in recalling information pertaining to healthy eating, the detrimental impact of smoking, appropriate handwashing procedures, and preventive measures for COVID-19 and the common cold. Notably, participants needed assistance to provide accurate answers, indicating some limitations in the effectiveness of the instrument in assessing the retrieval of health information among the surveyed population. Examples such as participants confusing details about the differences between COVID-19 and the common cold and expressing difficulty in remembering health-related instructions highlight the need for a comprehensive evaluation of retrieval aspects in health literacy assessment among Nepali teens.

*ID: 6 There is something different between Corona and the common cold. But I’ve heard that during the spread of the coronavirus, the way they spread and how to avoid them are the same on a talk show with a doctor on the radio. I forgot the name of this programme.*

*ID: 12 If I’m not feeling well, tell me to do this. do that at home. How can I remember everything? It is not taken care of. If I must go to the doctor, I go with my father. He’s talking to the doctor. He also buys and feeds me herbs. I’m not doing anything.*

### Judgment

Participants demonstrated superficial judgement in their responses, relying on simplistic assumptions when describing items. This approach may lead to a disregard for underlying health factors and reinforce stereotypes and biases related to body size and shape. These findings indicate participants’ surface-level awareness, highlighting the need for a deeper comprehension of the concepts presented in the items. Additionally, some of the participants acknowledged understanding the questions or terms but struggled to provide additional information due to insufficient knowledge.

ID_08: *Thin and fat body can be seen with the naked eye. Similarly, according to height and weight, very fat or very thin people can be distinguished.*

*ID_13: Health care is the act of cleaning our homes, neighbourhoods (tols), sewers, water, and the environment.*

### Response

Each participant read every question in the tool. We designed the survey instruments to meet the participants’ needs. Participants mentioned that the survey was well-printed, had a well-organised layout, and had clear and legible instructions and item text. However, they found minor spelling mistakes in the printed survey form. While most participants found the four-point Likert scale (“very difficult,” “difficult,” “easy,” “very easy,” and additional “don’t know”) appropriate, indicating their comfort with it. However, some adolescents needed clarification to understand it, indicating potential challenges with this response format. Additionally, participants tended to provide superficial and condensed answers. Some participants also showed low confidence in their responses, suggesting limitations in capturing genuine reflection. In our field observations and memos, we noted that participants responded rapidly, usually within about five seconds. These rapid reactions suggest a lack of deliberate consideration, often leading to unconscious, overlapping, insufficient, and inappropriate answers. Throughout the study, most participants consistently provided responses to the survey items. Overall, while the HLS-Child-Q22-NEP appears to capture certain aspects of health literacy, the findings indicate areas for improvement related to adolescents’ understanding and interpretation of items.

*ID 11: Don’t you know, many friends, even girls smoke cigarettes, eat tobacco, but they say no. I also started smoking in seventh grade, but I no longer do so.*

### Revision of survey items

The abovementioned findings highlighted the need for the re-evaluation and improvement of items, which is why the items were revised. Our main focus was to lower the complexity of language by incorporating simpler and more direct expressions that would be suitable for the intended participants. We replaced ambiguous terms with clearer alternatives. Also, the increase in the number of items from 22 to 24 required renaming the tool as HLS-Child-Q24-NEP (see Table 3).This iterative process was undertaken in order to optimize the tool’s effectiveness in assessing health literacy among Nepali teenagers.

**Table 3:** Modified HLS-Child-Q24 Items based on HLS-Child-Q15

S.N	Please tick (√) the appropriate option in the question below.	Very difficult	difficult	Easy	Very easy
1	It is easy or difficult for you to find out how to recover quickly when you have a common cold, or COVID.				
2	It is easy or difficult for you to find out what to do to prevent getting too fat or too thin.				
3	It is easy or difficult for you to find out how to rest properly.				
4	It is easy or difficult for you to find out which foods are healthy for you.				
5	It is easy or difficult for you to understand when and how you should take medicine when you are sick.				
6	It is easy or difficult for you to understand what your doctor and other health personnel say.				
7	It is easy or difficult to understand why you should sometimes go to a doctor or health personnel even though you are not ill.				
8	It is easy or difficult for you to understand the need for immunization.				
9	It is easy or difficult for you to understand what your parents tell about your health.				
10	It is easy or difficult for you to understand why you need to rest sometimes.				
11	It is easy or difficult for you to judge what helps a lot for you to stay healthy and what does not help much.				
12	It is easy or difficult for you to do what your parents tell you to do so that you can get well again.				
13	It is easy or difficult to you to take your medicine as you are suggested.				
14	It is easy or difficult for you to follow what you have learned in traffic education.				
15	It is easy or difficult for you to have a healthy diet.				
16	It is easy or difficult to judge what helps or does not help to get rid of a common cold or Corona.				
17	It is easy or difficult for you to judge the truth of what the doctor tells you to help you recover again.				
18	It is easy or difficult for you to trust and make decisions about the warnings given by the media and social networks about health risks.				
19	It is easy or difficult for you to judge what may happen to you later if you start smoking, alcohol, and drugs.				
20	It is easy or difficult to judge how your place of residence (neighborhood, district, road) makes you ill or helps you stay healthy				
21	It is easy or difficult for you to judge how your behavior (exercise and diet) is connected to your health.				
22	It is easy or difficult for you to decide when and why you need to do hand hygiene.				
23	It is easy or difficult for you to understand what your peers tell about your health.				
24	It is easy or difficult for you to do what your peers support you to do so that you can get well again.				

## Discussion

This study introduces the HLS-Child-Q22-NEP scale, the first comprehensive health literacy questionnaire designed to assess Nepali adolescents’ health literacy levels in the ninth grade. Through a rigorous tool development process, which included 13 CIs, we qualitatively tested all items to ensure their relevance and comprehension for Nepali-speaking adolescents. The findings identified strengths and areas needing refinement. This led to the revision of the instrument, the addition of two further items, and the renaming of the tool to HLS-Child-Q24-NEP. This study represents the first effort of its kind to adapt the HLS-Child-Q15 for use in Nepal. The cognitive interview results introduced four main themes: (i) comprehension, (ii) retrieval, (iii) judgement, and (iv) response.

The first theme, comprehension, refers to how survey respondents understand and interpret the questions or items presented to them in the survey [39]. This theme consists of two subcategories: item comprehension and word comprehension. Participants generally exhibited a positive acceptance of the survey questions, indicating a willingness to engage with the content. However, participants’ interpretations of items seemed to lean towards literal comprehension rather than the interpretation intended by the authors. This study identified two main issues affecting item comprehension. Firstly, participants encountered difficulties due to unfamiliarity and limited experience with certain vocabularies and words. Secondly, translation errors significantly challenged participants. Some items exhibited inconsistencies in sentence structure or syntax, including wording that was out of context, repetition, and errors in Nepali grammar.

Despite diligent translation efforts, achieving entirely appropriate translations for all participants proved challenging, primarily due to the diversity of the Nepali language. Furthermore, despite participants’ familiarity with certain words associated with the items, they needed assistance in comprehending specific actions and tasks related to those items. The subjective and context-dependent nature of participants’ perceptions regarding the complexity and ease of understanding of survey items underscored the necessity for tailored translation to enhance participants’ comprehension.

The study proposes that a revised translation, customised to the language nuances of the selected participants, could potentially improve their understanding of survey items. This suggestion aligns with the original study to develop the HLS-Child-Q15 [19], where pretest participants reported confusion regarding the meaning and relevance of items [20]. The findings underscore the importance of considering language and cultural factors in developing and translating health literacy assessment tools to ensure accurate and meaningful responses from participants.

In the second subcategory, word comprehension, participants encountered unfamiliar terms such as health promotion, health care, health determinants, health risk, and health warnings, resulting in incomplete responses. Additionally, participants’ misunderstandings of specific words, such as vaccine, media, and road safety, impact the scale’s ability to accurately measure the intended constructs. The study emphasises that the inability to retrieve and evaluate relevant information from memory may lead to inaccurate or incomplete responses, aligning with Tourneau’s concept [40]. Furthermore, the study suggests that the limited understanding of certain health concepts may be attributed to the state and scope of health education in Nepal, neglecting practical and critical aspects that link to everyday activities. The participants’ flawed understanding of concepts introduced in health education courses may result in incomplete interpretations [33]. The findings of the study conducted by Domanska et al. (2018) support the conclusion that adolescents lacked familiarity with certain terms in the questionnaire and required additional practical experience in healthcare interaction and disease prevention tasks [29]. Another comparable study concluded that using technical words in survey questionnaires could result in issues with understanding [42], although to a lesser extent than in this study. Identifying and addressing these issues related to questionnaires and respondents is recognized as crucial for increasing the accuracy and reliability of survey instruments. This, in turn, contributes to more robust conclusions and better-informed decisions. The evaluation of HLS-Child-Q22-NEP in relation to the word comprehension subcategory underscores the importance of refining the tool to enhance its effectiveness in assessing health literacy among Nepali adolescents.

The second theme, retrieval, relates to respondents needing to retrieve relevant information from their long-term memory, whether factual or attitudinal [43]. In this study, participants unanimously affirmed that most of the content within HLS-Child-Q22-NEP was relevant to their daily lives. However, a concerning pattern emerged as participants quickly read the items and promptly provided answers in a superficial and simplistic way [39]. The absence of a foundation in proven information or sound reasoning during the response process may have led to potential errors and misunderstandings. It was observed that participants had difficulty remembering, retaining, and applying the scientific health knowledge they had acquired or learned.

Even when participants were familiar with the words in some items, they exhibited a lack of complete understanding of the concepts, resulting in partial or incorrect recall [44] of the overall information. In certain instances, participants characterized the retrieved information with estimates and attitudes rather than current and factual information. This deviation from accurate recall may appear to be influenced by participants’ beliefs, values, feelings, and emotions. Participants seemed to forget or not memorising acquired knowledge, attributed to factors such as lack of dedication, infrequent practise, and reliance on guesswork rather than proper understanding, contributing to not memorising the information [43]. Social factors such as chronic poverty, low family literacy, inadequate infrastructure, deficient healthcare, gender and caste disparities, and poor socialisation may significantly affect participants’ understanding of the scale. This trend raises concerns about the reliability of responses and the depth of understanding among respondents.

Judgment (Theme 3) involves respondents looking at the items, absorbing the information, and using it to make informed decisions [40]. The results of the study shed light on a noteworthy observation: few participants demonstrated a lack of adequate judgment when responding to the items. The issue of misjudging responses during a recall task emerged as a potential challenge, and this misjudgment [43] could be attributed to incomplete information recall. In the context of health literacy, this signifies a critical concern as it suggests that the participants may struggle not only with recalling information accurately, as discussed in the retrieval theme, but also with employing sound judgement based on the available information.

The fourth theme of this study is response. As Tourneau [39] outlined, it involves two key aspects: answer editing and evaluating answer options. Answer editing involves respondents reviewing and modifying their answers before providing a final response, influenced by their overall survey experience. Considering answer options pertains to the respondent’s assessment of available choices and their decision-making process in selecting the most appropriate option. In our study, the observation of self-presentation responses, as described by Collins (2003) [43], emerged as a significant aspect. This points to the consistency of participants’ initial responses to survey items.

Overall, the study yielded promising results, indicating that the survey items were well-structured and that the provided options were suitable for the respondents. However, upon analysing participants’ responses to the items, we identified discrepancies between the generated and intended responses. These variations in responses arose from unconscious, overlapping, and insufficient responses. The unconscious category refers to instances where participants may have responded without conscious thought or deliberate consideration. Overlapping categories include items where the response aligns with multiple themes. Lastly, the inadequate category pertains to items whose responses did not fully address the desired aspects.

This study has identified issues related to the questionnaire and respondents. Questionnaire-related problems include the need for more precise instructions, translation issues, and the inclusion of some terms that respondents need help understanding. Similarly, among the issues related to respondents, they appear to have limited health knowledge and lack of experience, poor recall, provide answers based on guesswork, and have limited rationale for their answers. These factors can influence the responses given by the respondents. We used all these findings to revise the questionnaire, resulting in the HLS-Child-Q24-NEP.

## Limitations

This is the first study of its kind in Nepal. It provides initial insights on the applicability of the HLS-Child-Q22-NEP in Nepali school adolescents. This survey tool has many positive qualities: The revised questionnaire refers to adolescents’ daily lives, and the items’ printing, ordering, and layout are appropriate for the target group. Furthermore, the revised items use expressions that adolescents are familiar with. Therefore, the revised HLS-Child-Q24-NEP is tailored to a greater extent to the cognitive abilities and experiences of the target group. Considering the importance of this measure, we felt that additional cognitive interviews must be conducted to better understand the HLS-CHILD-Q24-NEP. Nonetheless, it has several limitations. Although we tried to ensure that the language and grammar were appropriate to our sample, it might not be suitable for Nepalese students from other backgrounds and locations. We have used the independent back-translation method to translate the English tool to Nepali. The translation outcome revealed some discrepancies, indicating translation errors in the target language version. Thus, further research is needed on the most appropriate translation for specific contexts.

The scale used in this study was developed and validated for primary school children aged 9–11 years in Germany. The age of the participants in the Nepali study was 13–19 years. That age-related context seems mismatched, although the HLS-Child-Q15 has been used in older cohorts and has proven valid and reliable in adolescents as old as 18 years. Further testing might be needed, as well as developing additional Nepali items that are particularly developed in and for the Nepali context together with Nepali adolescents.

This study has been conducted in a small sample, which means it is limited in scope and context. Therefore, the study’s results may not apply to a larger population and additional research is needed to draw more meaningful conclusions. While this study provides initial findings on the applicability of an adapted version of the HLS-Child-Q15 for Nepal, further qualitative research is needed to improve the applicability and understandability of the tool.

Finally, the study team found that participants might have felt embarrassed over not knowing many terms of the questionnaire, which might have prompted them not to give more in-depth answers.

## Conclusion

In this groundbreaking endeavour to adapt the HLS-Child-Q15 measure for adolescent health literacy in Nepal, the HLS-Child-Q22-NEP demonstrates promise in capturing specific facets of health literacy responses among Nepali adolescents. Nonetheless, the findings from the cognitive interview emphasize that the German HLS-Child-Q15 does not effectively align with the Nepali context, highlighting significant areas that require improvement. The difficulties encountered in understanding, recalling, judging, and responding offer valuable insights for enhancing the survey instrument, thereby enabling a more thorough evaluation of health literacy among Nepali adolescents using the HLS-Child-Q22-NEP.

The decision to reassess and revise survey items demonstrates a practical commitment to overcoming challenges. By addressing the refinement issues, the HLS-Child-Q22-NEP has the potential to become a more effective tool for comprehensively capturing health literacy among Nepali adolescents.

The study’s findings are pivotal in promoting better-informed health literacy interventions for Nepali adolescents. Following the study and adolescent interviews, the HLS-Child-Q22-NEP underwent further development, resulting in the HLS-Child-Q24-NEP, featuring additional and modified items. This thorough revision process positions the HLS-Child-Q24-NEP for further pretest studies, including a contextual pilot survey among Nepali adolescents, to ascertain its validity and reliability. This ongoing refinement underscores the commitment to advancing culturally sensitive and linguistically appropriate health assessments for adolescents in Nepal.

### Electronic supplementary material

Below is the link to the electronic supplementary material.

Supplementary Material 1

## Data Availability

The data used in this study are part of the first author’s Ph.D. study, and can be obtained upon reasonable request. We would be glad to provide you with access to the data if you send an email to khanalshanti100@gmail.com.

## Abbreviations

AHL: Adolescent Health Literacy
CI: Cognitive Interview
CLE: Cognitive Laboratory Environment
HLS-EU-Q47: European Health Literacy Survey Questionnaire
HLS-Child- Q15: Health Literacy Scale for Children
HLS-Child- Q 24- Nep: Nepali Version Health Literacy Scale for Children
