# Supplementary material for: Adolescents’ understanding of the Nepalese version of HLS-CHILD-Q15: qualitative pre-testing in ninth-graders
Source: BMC Public Health. 2024 Mar 19;24:851. doi: 10.1186/s12889-024-18329-9 (PMC10949603; doi:10.1186/s12889-024-18329-9)
Supplement: Supplementary file 1 — Supplementary Material 1 [file 12889_2024_18329_MOESM1_ESM.docx]

**Annex 1**

Table 1 *Adapted Nepali items based on the HLS-Child-Q15*

| S.N. | **How easy or difficult is it for you to…** | Very difficult | difficult | Easy | Very easy | don’t know |
| --- | --- | --- | --- | --- | --- | --- |
| **1** | find out how to recover quickly when you have a cold? |  |  |  |  |  |
| **2** | find out what you can do so that you don’t get too fat or too thin? |  |  |  |  |  |
| **3** | find out how you can best relax? |  |  |  |  |  |
| **4** | find out which food is healthy for you? |  |  |  |  |  |
| **5** | understand when and how you should take your medicine when you are ill? |  |  |  |  |  |
| **6** | understand what your doctor says to you? |  |  |  |  |  |
| **7** | understand why you sometimes need to see the doctor even though you are not ill? |  |  |  |  |  |
| **8** | understand why you need vaccinations? |  |  |  |  |  |
| **9** | understand what your parents tell you about your health? |  |  |  |  |  |
| **10** | understand why you need to relax sometimes? |  |  |  |  |  |
| **11** | judge what helps a lot for you to stay healthy and what does not help much? |  |  |  |  |  |
| **12** | do what your parents tell you to do so that you can get well again? |  |  |  |  |  |
| **13** | take your medicine in the way you’re told to? |  |  |  |  |  |
| **14** | stick to what you have learned in road safety lessons? |  |  |  |  |  |
| **15** | have a healthy diet? |  |  |  |  |  |
| **16** | judge what helps or does not help to get rid of a cold? |  |  |  |  |  |
| **17** | judge the truth of what the doctor tells you in order for you to get well again? |  |  |  |  |  |
| **18** | judge whether you can trust the media when they warn you about risks to your health? |  |  |  |  |  |
| **19** | judge whether what may happen to you later if you start smoking is true? |  |  |  |  |  |
| **20** | judge how where you live (neighborhood, district, street) is connected to your health? |  |  |  |  |  |
| **21** | judge how your behavior (exercise and diet) is connected to your health? |  |  |  |  |  |
| **22** | Decide when you need to wash your hands |  |  |  |  |  |
